# Illumination on “Reserving Phloem and Discarding Xylem” and Quality Evaluation of *Radix polygalae* by Determining Oligosaccharide Esters, Saponins, and Xanthones

**DOI:** 10.3390/molecules23040836

**Published:** 2018-04-05

**Authors:** Fan Yang, Huijuan Yu, Xin Chai, Siwei Peng, Junjun Yang, Dan Wu, Jie Du, Yuefei Wang

**Affiliations:** Tianjin State Key Laboratory of Modern Chinese Medicine, Tianjin University of Traditional Chinese Medicine, Tianjin 300193, China; yangfan_1992@foxmail.com (F.Y.); yuhuijuan_2017@126.com (H.Y.); chaixinphd@aliyun.com (X.C.); pswei3711441@163.com (S.P.); 13388055370@163.com (J.Y.); Angel_awind@163.com (D.W.); dj308888642@163.com (J.D.)

**Keywords:** *Radix polygalae*, UPLC-ESI-MS/MS, quantification, xylem, phloem

## Abstract

The root of *Polygala tenuifolia* Willd. or *Polygala sibirica* L. exhibits protective effects on the central nervous system and is frequently used to treat insomnia, amnesia, and other cognitive dysfunction. In our study, we studied nine bioactive compounds spanning oligosaccharide esters, saponins, and xanthones by using a sensitive, efficient, and validated method established on ultra-performance liquid chromatography coupled with triple quadrupole mass spectrometry. The quantified result of interesting compounds proved that accumulation of those compounds were found in phloem rather than in xylem. By taking the standardized result of nine compound contents into account, the “Spider-web” analytical result of xylem and phloem from *Radix polygalae* (RP) unveiled the rationality of RP’s classical use in clinic including discarding the xylem and reserving the phloem. Moreover, the remarkable variation was also revealed from the quantitative result of 45 samples with different diameters from the different origins, which did not significantly correlate with the variation of RP’s diameter. Our study could shed the light on the quality assessment of RP for further research and illustrate the scientific connotation of the processing method of “discarding the xylem and reserving the phloem”.

## 1. Introduction

Embodied by the Chinese pharmacopoeia (ChP, 2015), *Radix polygalae* (RP) is the dry root of *Polygala tenuifolia* Willd. or *Polygala sibirica* L. with the function of tranquilizing mind and promoting intelligence, which restores normal coordination between the heart and kidney by eliminating phlegm and reducing swelling [1]. According to the specific requirements of clinical medication, to enhance the function of dispelling phlegm and relieving cough, RP is usually processed with honey. To reduce toxicity and increase the curative effect, RP is concocted with licorice water decoction [2]. Oligosaccharide esters, saponins, and xanthones from RP were demonstrated to produce biological and pharmacological activity. Oligosaccharide esters exhibit marked biological activity on anti-dementia, brain protection, and anti-depression [3,4,5,6]. Saponins are the main pharmacological substances of RP, which exert the functions of tranquilizing and allaying excitement, expelling phlegm, and arresting coughing [7,8,9,10]. Xanthones have analgesic, antifungal, anti-cancer, and other effects [11,12,13].

As recorded by Compendium of *Materia Medica* [14] and Lei’s Treatise on Preparing Drugs [15], RP’s xylem is discarded to alleviate annoyance and RP’s phloem is reserved. According to our market survey of major Chinese herbal medicine markets, RP’s phloem is mostly used as medicine such as Heze (Shandong, China), Yuzhou (Henan, China), Zhangshu (Jiangxi, China), Guangzhou (Guangdong, China), and Bozhou (Anhui, China). Removal of xylem from RP is laborious, but is an indispensable procedure in the process of RP. From the view of distribution of active compounds between RP’s xylem and phloem, the systematic study should be undertaken to illustrate the scientific connotation of reserving RP’s phloem and discarding RP’s xylem. Additionally, emerging from the market survey is a picture that the bigger the diameter is, the more expensive the price is. The causal relationship should be delved between the diameter of RP’s phloem and accumulation of active compounds. Therefore, it is meaningful to answer these questions to guide the rational use of RP in the clinical setting by appraising the quantitative fluctuation of multiple bioactive constituents in collected samples.

Until now, various analytical methods were established to perform the quantitative and qualitative appraisal of bioactive constituents in RP, such as high performance liquid chromatography (HPLC) [16,17,18], ultra-performance liquid chromatography-quadrupole time-of-flight mass spectrometry (UPLC-Q-TOF) [19], and liquid chromatography-tandem mass spectrometry (LC-MS/MS) [20,21,22], which contributed to RP’s quality evaluation. For our study, there were some limitations in methods that cannot meet our study, such as qualitative study performed only [19,22], quantitative research on limited compounds [17,18,20,21], and longer analytical time [16,18].

In our study, by employing the UPLC-ESI-MS/MS method, nine compounds, sibiricose A5 (SA5), sibiricose A6 (SA6), sibiricaxanthone B (SXB), polygalaxanthone XI (PXXI), 3,6′-disinapoyl sucrose (DS), polygalacin D (PGD), tenuifolin (TF), polygalic acid (PGA), and senegenin (SG), were quantified in the collected RP’s samples within eight minutes using ginsenoside Rg1 (Rg1) as the internal standard (IS). Based on the visual analysis of the standardized result of the focused compounds’ contents and “Spider-web” mode, the results illustrated the scientific connotation of “discarding the xylem and reserving the phloem”, the diversity of compounds contents of RP from different origins and no correlation between bioactive compounds’ content and RP’s diameter. The aim of this paper was to provide the scientific foundation to remove xylem from RP as non-medicinal parts and the scientific evaluation of RP’s quality.

## 2. Results and Discussion

### 2.1. Optimization of UPLC-ESI-MS/MS Conditions and Sample Preparation

**Figure 1:**
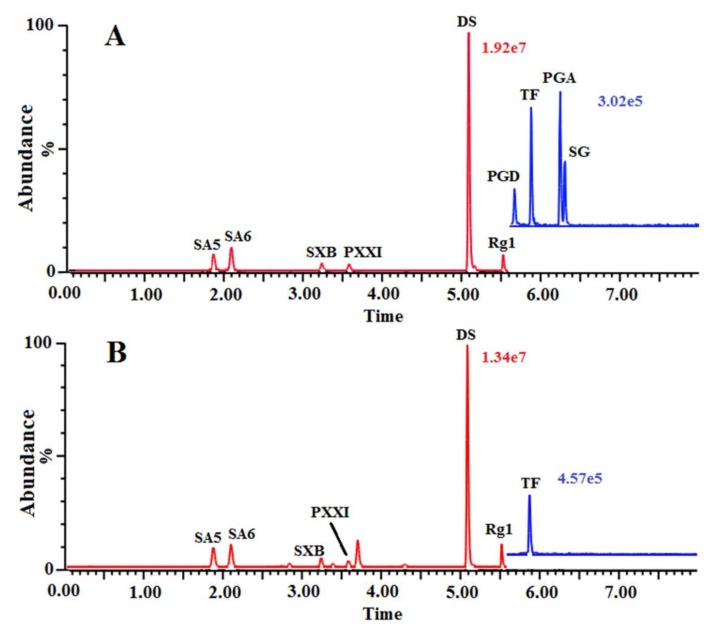
The representative MRM chromatograms of the reference standards solution (**A**) and sample solution (**B**).

Aiming at the high levels of sensitivity, resolution, and efficiency of the focused compounds, the chromatographic and MS/MS conditions were thoroughly investigated by optimizing the chromatographic parameters, spanning the gradient elution procedure and column temperature, and MS parameters, including the cone and capillary voltage, the source and desolvation temperature, and the desolvation and cone gas flow. The optimized parameters of compounds in MRM mode for UPLC-MS/MS analysis were shown in Table 1. The representative chromatograms were shown in Figure 1.

Subsequently, the sample preparation was subjected to strict screening including the extraction solvent (the different methanol acqueous solution with 25%, 50%, 75%, and 100% methanol), the extraction volume (10 mL, 25 mL, 50 mL, and 100 mL) and the sonicated time (10 min, 20 min, 30 min, and 40 min). It was revealed by Figure S1 that the extraction volume and sonicated time had more influence on the extracting efficiency of the compounds than the extraction solvent. The extracting parameters were optimized in our study.

### 2.2. Methodological Validation of the Quantitative Analysis

The developed UPLC-MS/MS method was validated for quantitative analysis of the targeted compounds. The detailed results were summarized in Table 2. All calibration curves showed good linearity (*r* ≥ 0.9959) across the tested ranges of the targeted compounds. At the same time, the overall LOD and LOQ values were less than 0.40 ng/mL and 1.60 ng/mL, respectively. Other than the inter-day precisions of SXB and PXXI, the RSDs of intra-day and inter-day precisions, repeatability, and stability for the nine analytes were all below 5.80%. The mean recovery rates of the bioactive compounds ranged from 96.92% to 106.2% with RSD value below 6.73%. All these results indicated that this UPLC-MS/MS method was reliable and appropriate for simultaneously determining the nine bioactive compounds of RP.

### 2.3. Distribution Analysis of Interesting Compounds in RP’s Xylem and Phloem

According to the book, Lei’s Treatise on Preparing Drugs, a classical masterpiece of Traditional Chinese Medicine (TCM), RP’s phloem was usually employed as medicinal use after removing the xylem as its non-medicinal parts [15]. From the distribution of bioactive compounds in RP’s xylem and phloem, we wanted to interpret the scientific basis of “discarding the xylem and reserving the phloem”.

$C_{A}^{M}$ and ${C{}^{\prime }}_{A}^{M}$ were assigned as the content of SA5, SA6, SXB, PXXI, TF, DS, PGA, SG, and PGD in RP’s xylem and phloem quantified, respectively. A was designated as the different batches of RP (BZ-1~BZ-7, HZ-15, HZ-16, and ZS-7). M represented the different compounds including SA5, SA6, SXB, PXXI, TF, DS, PGA, SG, and PGD. The RP’s xylem and phloem had different weights and accounted for the different mass ratio of the root (xylem + phloem), which were labeled as $X_{A}$ and $P_{A}$, separately. The moisture content of xylem and phloem were labeled as $W_{A}$ and ${W^{\prime }}_{A}$, respectively. Considering that the different moisture content and mass ratio of RP’s xylem and phloem would affect the distribution ratio of bioactive compounds in xylem and phloem, the standardized content of bioactive compounds in the corresponding RP’s xylem and phloem were obtained and marked as $D_{A}^{M}$ and ${D{}^{\prime }}_{A}^{M}$, respectively.

(1) $$C_{A}^{M}\times X_{A}\times (1-W_{A})=D_{A}^{M},$$

(2) $${C{}^{\prime }}_{A}^{M}\times P_{A}\times (1-{W^{\prime }}_{A})={D{}^{\prime }}_{A}^{M},$$

$D_{A}^{M}$ and ${D{}^{\prime }}_{A}^{M}$ were calculated using the above formula. Then the *t*-test of the standardized content of bioactive compounds between RP’s xylem and phloem was carried out. As displayed in Figure S2, the distribution of bioactive compounds (except for PGA, SG, and PGD undetected) in phloem and xylem was significantly different (*P* < 0.001). The bioactive compounds in phloem had a great amount exceeding from 3 to 192 times higher than that in xylem. It can be deduced that the processing method of RP, “discarding the xylem and reserving the phloem”, is meant to remove non-medicinal parts. Our study result was coincident with the reported research, which performed the comparison between the RP’s xylem and phloem by quantifying polygalaxanthone Ш (PXШ), TF, and DS [23].

In order to display the great disparity between RP’s xylem and phloem in a more visual and monolithic style, the “Spider-web” mode was employed, which was developed by our team [24,25] by allowing for the standardized content of SA5, SA6, SXB, PXXI, TF, DS, PGA, SG, and PGD in RP’s xylem and phloem. For the sake of eliminating the influence of the different range of $D_{A}^{M}$ and ${D{}^{\prime }}_{A}^{M}$ value on the statistical results, the normalization method was applied in this study. Because of the higher content of active compounds in RP’s phloem compared to that in xylem, the highest value of ${D{}^{\prime }}_{A}^{M}$ was tagged as ${D{}^{\prime }}_{A}^{M}$ (max). All the $D_{A}^{M}$ and ${D{}^{\prime }}_{A}^{M}$ were divided by the corresponding ${D{}^{\prime }}_{A}^{M}$ (max) respectively, which were accordingly marked as $E_{A}^{M}$ and ${E{}^{\prime }}_{A}^{M}$. The equations are listed below.

(3) $$E_{A}^{M}=D_{A}^{M}/{D{}^{\prime }}_{A}^{M}(\max),$$

(4) $${E^{\prime }}_{A}^{M}={D{}^{\prime }}_{A}^{M}/{D{}^{\prime }}_{A}^{M}(\max),$$

**Figure 2:**
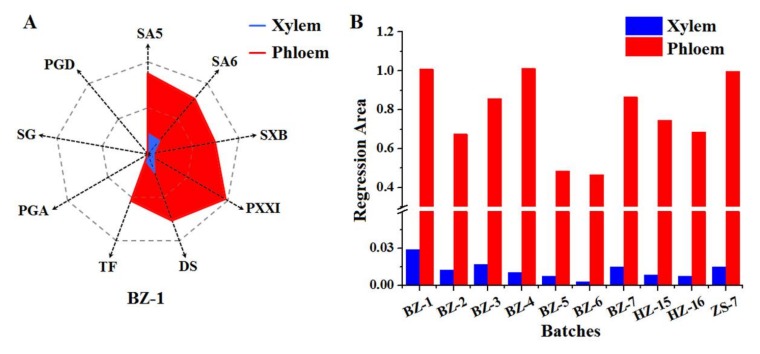
The representative “spider-web” mode of RP’s xylem and phloem established by taking the standardized content of nine compounds into account (**A**) and the histogram of shade area calculated from “Spider-web” mode of xylem and phloem from the different batches, respectively (**B**).

Taking the sample (BZ-1) as an example, the normalized values ($E_{A}^{M}$ and ${E{}^{\prime }}_{A}^{M}$) of SA5, SA6, SXB, PXXI, TF, DS, PGA, SG, and PGD were obtained by using the equations, which were respectively employed as nine dimensions of the “Spider-web” mode for xylem and phloem and labeled as *p_i_* (*i*, 1~9) and *p*′*_i_* (*i*, 1~9), respectively. The “Spider-web” mode of xylem and phloem were constructed as shown in Figure 2A. The shade areas were calculated by the equations below. The angles between two dimensions in the “Spider-web” mode for xylem and phloem were titled as α and α′, respectively. The shade area of xylem’s “Spider-web” mode was 0.029 while that of phloem’s “Spider-web” mode was 1.009.

(5) $$S=\frac{1}{2}\sin\alpha (\sum_{i=1}^{n-1}p_{i}\times p_{i+1}+p_{n}\times p_{1}),$$

(6) $$S^{\prime }=\frac{1}{2}\sin\alpha^{\prime }(\sum_{i=1}^{n-1}{p^{\prime }}_{i}\times {p^{\prime }}_{i+1}+{p^{\prime }}_{n}\times {p^{\prime }}_{1}),$$

Similarly, the result of shade area of the “Spider-web” mode fitted by the standardized content of nine compounds from RP’s xylem and phloem of other batches was shown in Figure 2B. It was observed that the shade area of the “Spider-web” mode of RP’s phloem was 35~179 times as high as that of corresponding xylem. In the light of visual and integral “Spider-web” mode, it easily displayed the disparity of bioactive compounds’ distribution between xylem and phloem, which deeply proved the wisdom of RP’s processing method by discarding the xylem and reserving the phloem.

### 2.4. The Heat Map Analysis of Interesting Compounds in the Samples from Different Origins

The obvious fluctuation of bioactive compounds’ content in RP’s phloem from forty-five origins was displayed by the validated UPLC-MS/MS analytical method. As shown in supporting information Table S1, the total content of oligosaccharide esters ranged from 422.6 to 11672.3 μg/g covering SA5, SA6, and DS. The total content of xanthones varied from 928.9 to 2236.7 μg/g spanning SXB and PXXI. The content of saponins was between 13.9 and 69.5 μg/g including TF, SG, PGA, and PGD, respectively. In particular, except for GZ-4 and GZ-6 from which DS was not detected, DS was the predominant compound in the collected samples, which was proven to possess an anti-depression pharmacological property [5,26]. The ChP stipulated that the content of DS should be ≥0.50% in the crude RP and ≥0.30% in the licorice-processed RP while the content of TF should be both ≥2.0% for the crude and licorice-processed RP [1]. In the collected samples, only 24.4% of the crude RP and 75.0% of the licorice-processed RP qualified for the DS standard released by the ChP. As for TF treated by our established method, none of the crude and licorice-processed RP reached the ChP standard, which was extremely low in the condition of the “original state”. Actually, published by the ChP, the alkaline hydrolysis method was adopted to determine the transformed TF, which transformed TF-conjugates into TF. Our study focused on the original TF rather than the transformed TF while the ChP targeted the transformed TF. PGA, SG, and PGD were not detected in our study.

**Figure 3:**
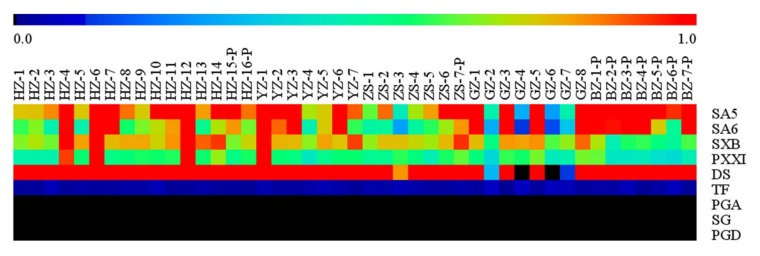
The heat map of relative concentration of the targeted compounds in forty-five samples.

In order to project the striking variety of the interesting compounds’ content in a visual mode, a heat map was used to display the trends with regard to the relative concentration of bioactive compounds in the different RP’s phloem. Looking at Figure 3, a red color sub-boxes expressed that these samples contained higher levels of the bioactive compounds when compared with the other samples and a blue-green sub-boxes indicated that these samples contained lower levels of the bioactive compounds. The heat map showed that the content of DS, PXXI, and TF was relatively stable, but that of SA5, SA6, and SXB varied remarkably.

### 2.5. The Correlation between the Bioactive Compounds’ Content and RP’s Diameter 

With the advent of exhaust of RP’s wild resources, the enormous need in the clinical setting increasingly spawned artificial planting of RP. Through our market survey of major Chinese herbal medicine markets, the positive association was unveiled between the RP’s price and specifications. Given that, it is necessary to make a systematic investigation about the correlation between the bioactive compounds’ content and RP’s diameter.

Upon the quantitative data, a correlation coefficient was used to analyze the association of accumulation of the bioactive compounds with the RP’s diameter, which was listed as follows. In order to perform overall analysis, the RP’s diameter was employed to undertake the analysis of association with the content of compounds derived from the different type including oligosaccharide esters (Y1) spanning SA5, SA6, and DS, xanthones (Y2) comprised of SXB and PXXI, saponins (Y3) that contain TF, SG, PGD, and PGA, and total content of the detected compounds (Y4), respectively. Here *r* was given as the linear correlation coefficient, *x* was specified as RP’s diameter, *y* was respectively defined as Y1, Y2, Y3, and Y4, and *i* was designated as batch number (*i*, 1~45). The total information of RP’s diameter and content of bioactive compounds was listed in supporting information in Tables S1 and S2.

(7) $$r=\frac{n\sum_{i=1}^{n}x_{i}y_{i}-\sum_{i=1}^{n}x_{i}\sum_{i=1}^{n}y_{i}}{\sqrt{n\sum_{i=1}^{n}{\left(x_{i}\right)}^{2}-{\left(\sum_{i=1}^{n}x_{i}\right)}^{2}}\sqrt{n\sum_{i=1}^{n}{\left(y_{i}\right)}^{2}-{\left(\sum_{i=1}^{n}y_{i}\right)}^{2}}},$$

**Figure 4:**
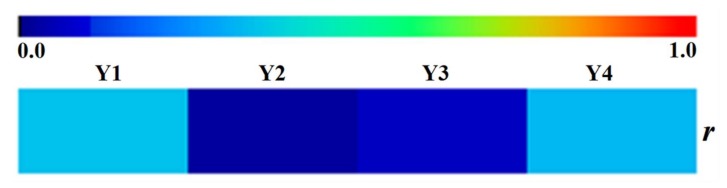
The heat map of the correlation coefficients between the diameter of RP and content of oligosaccharide esters, xanthones, saponins and total focused compounds, respectively.

As illustrated in Figure 4, it was clearly evident that the correlation coefficients were unsatisfactory between RP’s diameter and Y1, Y2, Y3, or Y4, all of which were below 0.345. This result pointed out that the diameter of RP has no obvious correlation with accumulation of bioactive compounds regardless of whether it is oligosaccharide esters, xanthones, and saponins or the all compounds in our study.

## 3. Materials and Methods 

### 3.1. Reagents and Materials

Except for DS with purity at about 95.3%, the reference standards with purity above 98% including SA5, SA6, SXB, PXXI, PGD, TF, PGA, and SG were obtained from Chengdu Pufei De Biotech Co., Ltd (Sichuan, China).

Acetonitrile and methanol (LC-MS grade) were purchased from Sigma-Aldrich Co., Ltd (St. Louis, MO, USA). Formic acid and dimethyl-sulfoxide (DMSO) were purchased from Meridian Medical Technologies (MREDA, New York, NY, USA). Water for UPLC analysis was purified by a Milli-Q water purification system (Millipore, Massachusetts, MA, USA).

Forty-five batches of RP collected from Chinese herbal medicine markets of Heze (Shandong, China), Yuzhou (Henan, China), Zhangshu (Jiangxi, China), Guangzhou (Guangdong, China), and Bozhou (Anhui, China), were labeled as HZ-1~HZ-16, YZ-1~YZ-7, ZS-1~ZS-7, GZ-1~GZ-8, and BZ-1~BZ-7, respectively. Except for BZ-1~BZ-7, HZ-15, HZ-16, and ZS-7, xylems of other batches of RP have been removed. The total information was shown in supporting information in Table S2. RPs with xylem and phloem (BZ-1~BZ-7, HZ-15, HZ-16, and ZS-7) were separated into xylem and phloem by ourselves, which were assigned as BZ-1-P, BZ-1-X, and so on. All plant materials were authenticated by Prof. Tianxiang Li and deposited at the herbarium in the Tianjin State Key Laboratory of Modern Chinese Medicine (Tianjin, China).

### 3.2. Standard Solution Preparation

Ginsenoside Rg1, the internal standard, was employed in this study. Accurately weighed standards were dissolved in 50% methanol aqueous solution (*v*/*v*) with 10% DMSO to prepare the stock solution for SA5 at 1.042 mg/mL, SA6 at 1.04 mg/mL, SXB at 1.035 mg/mL, PXXI at 1.036 mg/mL, DS at 1.054 mg/mL, PGD at 1.025 mg/mL, TF at 1.042 mg/mL, PGA at 1.054 mg/mL, SG at 1.03 mg/mL, and Rg1 (IS) at 1.052 mg/mL, respectively. For plotting the calibration curve, the working standard solution containing all the nine standards was prepared by serially diluting the mixed standards solution with 50% methanol to obtain eight different concentrations with the final concentration of Rg1 (IS) at 52.6 ng/mL. The linear interval tested ranged from 8.14 to 1042 ng/mL for SA5, from 8.13 to 1040 ng/mL for SA6, from 4.04 to 517.5 ng/mL for SXB, from 4.05 to 518 ng/mL for PXXI, from 41.17 to 5270 ng/mL for DS, from 0.20 to 25.63 ng/mL for PGD, from 0.20 to 26.05 ng/mL for TF, from 0.21 to 26.35 ng/mL for PGA, and from 0.20 to 25.75 ng/mL for SG. The prepared solution was stored at 4 °C until it underwent analysis.

### 3.3. Sample Preparation

Each accurately weighted powder (about 0.2 g) was sonicated separately by ultrasonicator (600 W, 50 Hz) using 25 mL 50% methanol aqueous solution (*v*/*v*) containing 52.6 ng/mL Rg1 (IS) for 30 minutes and then cooled to room temperature. The extracted solution was centrifuged at 14,000 rpm for 10 min and the supernatant was filtered through 0.22 µm syringe filter. The filtrate was further diluted 100 fold with 50% aqueous methanol solution (*v*/*v*) containing 52.6 ng/mL Rg1 (IS) and then analyzed by injecting 2 μL aliquot into the UPLC-ESI-MS/MS system.

### 3.4. UPLC-MS/MS Conditions

Equipped with binary solvent manager, sample manager, and column oven, ACQUITY^TM^ UPLC I-Class system (Waters, Milford, MA, USA) was employed to perform chromatographic analysis, which was controlled by MassLynx V4.1 software (Waters, Milford, MA, USA). Carried on ACQUITY^TM^ UPLC BEH C18 column (2.1 mm × 50 mm, 1.7 μm) at 40 °C, the chromatographic separation was accomplished by mobile phase consisting of 0.1% formic acid aqueous solution (*v*/*v*) (A) and acetonitrile (B) in a gradient elution, which was conducted as follows: 0–2 min, 5–10% B; 2–3 min, 10–14% B; 3–4 min, 14% B; 4–5 min, 14–25% B; 5–6 min, 25–70% B; 6–7 min, 70% B; 7–8 min, 70–95% B. The flow rate of mobile phase was set at 0.5 mL/minute and the injection volume was 2 μL.

The UPLC system was coupled to Waters Xevo TQ-S triple quadrupole mass spectrometer (Waters, Milford, MA, USA) equipped with an electrospray ionization source operating in the negative ion mode. The optimized parameters were as follows: capillary voltage at −2.0 kV, source temperature at 150 °C, desolvation temperature at 500 °C, desolvation gas flow at 1000 L/h, cone gas flow at 150 L/h, and nebulizer gas flow at 7.0 bar. The multiple reaction monitoring (MRM) acquisition mode was performed to detect the focused compounds after optimizing the parameter of each compound, such as cone voltage and collision energy. The cone voltage and collision energy of the detected compounds were listed in Table 1. All the data were acquired and processed by MassLynx V4.1 software (Waters, Milford, MA, USA).

### 3.5. Method Validation

The UPLC-MS/MS method established in this study was validated for linearity, LOD, LOQ, precision (intra- and inter-day), stability, repeatability, and recovery test. Calibration curves were constructed based on the weighted (1/*x*^2^) linear regression of the peak area ratios of the analytes to IS (*y*) versus the corresponding concentrations (*x*) of eight standard solutions at different concentrations in triplicate. The LOD and LOQ were determined by a signal-to-noise ratio (S/N) at about 3 and 10 using standard solution, respectively. The intra-day and inter-day precisions were conducted with six replicate injections of the same sample solution performed on the same day and on three consecutive days, respectively. To confirm the repeatability, six replicates of the same sample were prepared and analyzed by the established procedure. The stability of the sample solution stored in the UPLC autosampler at 4 °C was investigated by repeated injection at 0, 2, 4, 8, 10, and 12 h, respectively. A recovery test was used to further evaluate the accuracy of the analytical method. Accurate amounts of the nine bioactive compounds were added to 0.1 g sample powder in sextuplicate, whose sample solutions were prepared and analyzed with the method described above.

### 3.6. Data Analysis

The “Spider-web” mode was made by Excel 2016.Lnk software (2016, Microsoft Corporation, Redmond, WA, USA). The heat map was displayed by using the MultiExperiment Viewer software (MeV 4.9.0, Dana-Farber Cancer Institute, Boston, MA, USA). The histogram was plotted using GraphPad Prism 5.01 software (Graphpad software Inc., La Jolla, CA, USA).

## 4. Conclusions

The validity of traditional usage of RP by discarding the xylem and reserving the phloem was proven by the visual disparity of the standardized content of compounds in xylem vs. phloem and the constructed “Spider-web” mode by taking the distributional disparity of nine detected compounds between xylem and phloem into account. On grounds of the content survey of the determined compounds in the collected samples, the quality of RP’s phloem was ragged, which did not depend on the diameter of RP. This study shed light on the processing technology and the comprehensive quality evaluation of RP.

## Figures and Tables

**Table 1:** The detecting parameters of compounds in MRM mode for LC-MS/MS analysis.

Compound	Formula	*t_R_* (min)	MRM Transitions	Cone Voltage (V)	Collision Energy (eV)
SA5	C_22_H_30_O_14_	1.87	517.00→174.92	68	20
SA6	C_23_H_32_O_15_	2.09	547.02→204.96	66	24
SXB	C_24_H_26_O_14_	3.23	536.97→314.89	82	32
PXXI	C_25_H_28_O_15_	3.57	567.05→271.77	88	48
DS	C_34_H_42_O_19_	5.08	753.07→204.96	90	36
PGD	C_57_H_92_O_27_	5.67	1207.36→469.04	90	52
TF	C_36_H_56_O_12_	5.86	679.15→455.12	78	24
PGA	C_29_H_44_O_6_	6.20	487.16→469.13	76	30
SG	C_30_H_45_ClO_6_	6.24	535.20→481.09	56	32
Rg1 (IS)	C_42_H_72_O_14_	5.52	845.33→799.20	70	22

**Table 2:** Regression equations, LODs, LOQs, intra-day and inter-day precisions, repeatability, stability, and recovery test for the nine detected compounds of RP.

Compound	Regression Equations (*n* = 3)	*r*	Linear Range (ng/mL)	LOD (ng/mL)	LOQ (ng/mL)	Intra-Day (RSD, %; *n* = 6)	Inter-Day (RSD, %; *n* = 3)	Repeatability (*n* = 6)		Stability	Recovery (*n* = 6)	
								Mean (µg/g)	RSD (%)	RSD (%)	Recovery (%)	RSD (%)
SA5	*y* = 15.018*x* − 0.035	0.9999	8.14–1042	0.26	0.65	2.08	5.80	1536.17	2.44	2.00	103.3	5.35
SA6	*y* = 20.351*x* + 0.0977	0.9998	8.13–1040	0.26	0.65	1.54	3.42	1112.03	1.74	1.59	96.92	3.46
SXB	*y* = 10.741*x* + 0.0485	0.9995	4.04–517.5	0.32	0.81	2.19	8.02	661.48	2.26	1.15	97.73	5.40
PXXI	*y* = 9.5114*x* + 0.0385	0.9996	4.05–518	0.32	0.81	2.89	7.20	499.65	2.23	2.52	99.46	3.18
DS	*y* = 22.095*x* + 2.4691	0.9959	41.17–5270	0.13	0.33	2.26	3.26	5394.47	2.56	2.56	97.18	2.17
TF	*y =* 26.907*x +* 0.0071	0.9998	0.20–26.05	0.10	0.41	2.37	4.62	30.92	3.97	5.01	106.2	6.12
PGA	*y* = 33.341*x* + 0.0057	0.9999	0.21–26.35	0.10	0.41	–	–	–	–	–	104.2	2.81
SG	*y* = 18.072*x* + 0.003	0.9996	0.20–25.75	0.20	0.80	–	–	–	–	–	104.7	6.73
PGD	*y* = 9.7145*x* + 0.0009	0.9996	0.20–25.63	0.40	1.60	–	–	–	–	–	97.59	3.28

‘–’, Undetected.

## Supplementary Materials

The following are available online. Figure S1: The influence factors, the extracting solvent (**A**), extracting time (**B**), and extracting volume (**C**) on the extracting efficiency of nine targeted compounds from RP. Figure S2: The standardized content of bioactive compounds in RP’s xylem and phloem (*, *p* < 0.001), Table S1: Contents of 9 analytes in RP samples (mean ± SD, μg/g). Table S2: The information of RP samples used in this study.

## References

[1] National Commission of Chinese Pharmacopoeia (2015). Pharmacopoeia of the People’s Republic of China.

[2] Ren M.X., Guo J.M. (2002). Handbook of Modern Chinese Medicine Processing.

[3] Naito R., Tohda C. (2006). Characterization of anti-neurodegenerative effects of *Polygala tenuifoliain* A β (25–35)-treated cortical neurons. Biol. Pharm. Bull..

[4] Ikeya Y., Takeda S., Tunakawa M., Karakida H., Toda K., Yamaguchi T., Aburada M. (2004). Cognitive improving and cerebral protective effects of acylated oligosaccharides in *polygala tenuifolia*. Biol. Pharm. Bull..

[5] Liu P., Hu Y., Guo D.H., Wang D.X., Tu H.H., Ma L., Xie T.T., Kong L.Y. (2010). Potential antidepressant properties of radix polygalae (Yuan Zhi). Phytomedicine.

[6] Hu Y., Liao H.B., Dai-Hong G., Liu P., Wang Y.Y., Rahman K. (2010). Antidepressant-like effects of 3,6′-disinapoyl sucrose on hippocampal neuronal plasticity and neurotrophic signal pathway in chronically mild stressed rats. Neurochem. Int..

[7] Cao Q., Jiang Y., Cui S.Y., Tu P.F., Chen Y.M., Ma X.L., Cui X.Y., Huang Y.L., Ding H., Song J.Z. (2016). Tenuifolin, a saponin derived from Radix Polygalae, exhibits sleep-enhancing effects in mice. Phytomedicine.

[8] Kim K.S., Lee D.S., Bae G.S., Park S.J., Kang D.G., Lee H.S., Oh H., Kim Y.C. (2013). The inhibition of JNK MAPK and NF-*κ*B signaling by tenuifoliside A isolated from *Polygala tenuifolia* in lipopolysaccharide-induced macrophages is associated with its anti-inflammatory effect. Eur. J. Pharmacol..

[9] Chung I.W., Moore N.A., Oh W.K., O’Neill M.F., Ahn J.S., Park J.B., Kang U.G., Kim Y.S. (2002). Behavioural pharmacology of polygalasaponins indicates potential antipsychotic efficacy. Pharmacol. Biochem. Behav..

[10] Peng W.D., Xu S.P. (1998). Antitussive and expectorant effects of four saponins isolated from *Polygala tenuifolia* Willd. Chin. Pharm. J..

[11] De Campos R.O., Santos A.R., Vaz Z.R., Pinheiro T.R., Pizzolatti M.G., Cechinel Filho V., Delle Monache F., Yunes R.A., Calixto J.B. (1997). Antinociceptive properties of the hydroalcoholic extract and preliminary study of a xanthone isolated from *Polygala cyparissias* (Polygalaceae). Life Sci..

[12] Marston A., Hamburger M., Sordat-Diserens I., Msonthi J.D., Hostettmann K. (1993). Xanthones from *Polygalae nyikensis*. Phytochemistry.

[13] Zhang M., Wen J.M., Li W.K., Mai N.Q. (2001). Study on telomerase activity during induction of axon-like and dendrite-like processes in neuroblastoma cells. Chin. J. Nerv. Ment. Dis..

[14] Li S.Z. (1982). Compendium of Materia Medica.

[15] Lei X. (1985). Lei’s Treatise on Preparing Drugs.

[16] Li J., Dong X.B., Jiang Y., Gao Q.T., Jiang Z.Y., Cheung A.W., Duan R., Dong T.T., Tu P.F., Tsim K.W. (2007). Simultaneous determination of phenols in Radix Polygalae by high performance liquid chromatography: Quality assurance of herbs from different regions and seasons. J. Sep. Sci..

[17] Li J., Dong X.B., Jiang Y., Dong T.X., Tu P.F., Zhan H.Q. (2007). HPLC determination of total saponins in Radix Polygalae. Chin. J. Pharm. Anal..

[18] Chen S.L., Lin L.L., Chen S.B., Yang D.J., Yang J.S., Xiao P.G. (2005). Quantitative determination of nine xanthones in *Polygala caudata* and fingerprinting of Polygala L. by HPLC. J. Liq. Chromatogr. Relat. Technol..

[19] Ling Y., Li Z.X., Chen M.C., Sun Z.L., Fan M.S., Huang C.G. (2013). Analysis and detection of the chemical constituents of Radix Polygalae and their metabolites in rats after oral administration by ultra-high-performance liquid chromatography coupled with electrospray ionization quadrupole time-of-flight tandem mass spectrometry. J. Pharm. Biomed. Anal..

[20] Yang G.H., Cheng H., Huang Q., Yan Z.H., Yang W.L., Chen H.F., Yuan J.B. (2014). Determination of Polygalaxanthone Ⅲ and 3,6′-disinapoyl sucrose in *Polygala tenuifolia* by HPLC-MS/MS. Chin. J. Exp. Tradit. Med. Formulae.

[21] Lin L.F., Yan L., Zhang H., Li X.C., Zhang J., Dou H.R., Shen M.R., Yin X.B., Qu C.H., Ni J. (2014). Simultaneous analysis of polygala acid, senegenin and 3,6′-disinapoyl sucrose in rat plasma by liquid chromatography-tandem mass spectrometry: application to a pharmacokinetic study after oral administration. Biomed. Chromatogr..

[22] Dan W., He J.R., Jiang Y.M., Yang B. (2015). Quality analysis of *Polygala tenuifolia* root by ultrahigh performance liquid chromatography-tandem mass spectrometry and gas chromatography-mass spectrometry. J. Food Drug Anal..

[23] Liu Y.F., Peng D.Y., Yang X.J., Shi T.X., Jing Y., Tu P.F. (2012). Comparison of the chemical constituents and pharmacological activities between the cortexes and the roots of *Polygala tenuifolia*. Chin Pharm. J..

[24] Yang J., Jiang Z.Z., Chai X., Zhao B.C., Zhao X.P., Wang Y.F. (2016). Discriminant analysis of “Q-Markers” of traditional Chinese medical injections—Taking Dan Hong injection as a model. Mod. Tradit. Chin. Med. Mater. Medica-World Sci. Technol..

[25] Jiang Z.Z., Wang Y.F. (2016). A pattern of hierarchical progression for quality standard of Chinese materia medica based on “herbal origin-material basis-quality markers-quality control method”. Chin. Tradit. Herb. Drugs.

[26] Hu Y., Li J., Liu P., Chen X., Guo D.H., Li Q.S., Rahman K. (2012). Protection of SH-SY5Y neuronal cells from glutamate-induced apoptosis by 3,6′-disinapoyl sucrose, a Bioactive Compound Isolated from Radix Polygala. J. Biomed. Biotechnol..

