# Supplementary material for: Illumination on “Reserving Phloem and Discarding Xylem” and Quality Evaluation of Radix polygalae by Determining Oligosaccharide Esters, Saponins, and Xanthones
Source: Molecules. 2018 Apr 5;23(4):836. doi: 10.3390/molecules23040836 (PMC6017119; doi:10.3390/molecules23040836)
Supplement: Supplementary file 1 [file molecules-23-00836-s001.pdf]

Supplementary Materials to:

Illumination on “reserving phloem and discarding xylem” and quality evaluation of *Radix polygalae* by determining oligosaccharide esters, saponins and xanthones

Fan Yang†, Huijuan Yu†, Xin Chai, Siwei Peng, Junjun Yang, Dan Wu, Jie Du, Yuefei Wang*

Tianjin State Key Laboratory of Modern Chinese Medicine, Tianjin University of Traditional Chinese Medicine, Tianjin 300193, China; yangfan_1992@foxmail.com (F.Y.); yuhuijuan_2017@126.com (H.Y.); chaixinphd@aliyun.com (X.C.); pswei3711441@163.com (S.P.); 13388055370@163.com (J.Y.); Angel_awind@163.com (D.W.); dj308888642@163.com (J.D.); wangyuefei_2006@hotmail.com (Y.W.)

* Correspondence: wangyuefei_2006@hotmail.com; Tel.: +86-22-2738-6453

† These authors contributed equally to this work.

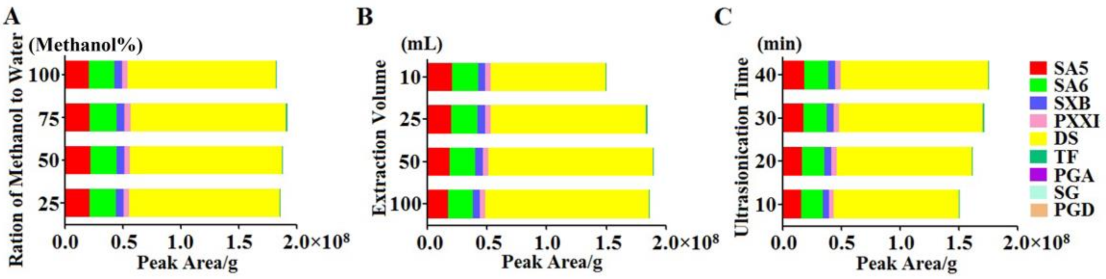

Figure S1. The influence factors, the extracting solvent (**A**), extracting time (**B**) and extracting volume (**C**), on the extracting efficiency of nine targeted compounds from RP.

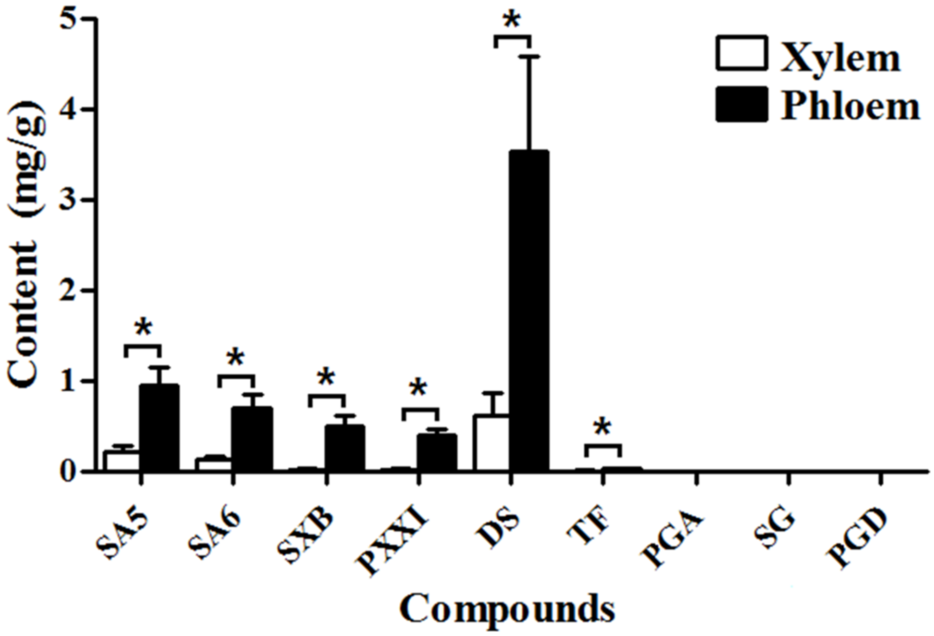

Figure S2. The standardized content of bioactive compounds in RP's xylem and phloem (*, *P*<0.001)

Table S1. Contents of 9 analytes in RP samples (mean ± SD, μg/g)

Sample	SA5	SA6	SXB	PXXI	DS	TF	PGA	SG	PGD
HZ-1	772.9±10.1	618.6±13.5	679.8±15.1	497.4±11.9	3248.8±20.5	20.5±0.8	–	–	–
HZ-2	776.8±6.9	678.6±22.5	718.1±13.5	491.0±18.7	3526.9±129.9	22.3±0.8	–	–	–
HZ-3	845.3±28.9	482.6±5.8	666.4±5.3	491.2±15.9	1597.9±65.8	51.5±2.0	–	–	–
HZ-4	1837.1±57.8	980.5±2.6	1095.8±10.6	924.8±40.1	3858.9±91.9	19.7±1.3	–	–	–
HZ-5	770.1±11.9	489.0±16.3	817.8±14.6	578.5±8.9	1420.9±56.6	28.1±1.4	–	–	–
HZ-6	2268.4±21.1	1121.8±5.7	1100.9±10.5	987.0±24.7	3524.3±60.8	21.9±0.7	–	–	–
HZ-7	1401.5±19.4	1082.2±15.6	791.4±13.2	550.1±16.7	4526.2±89.4	26.2±1.1	–	–	–
HZ-8	871.4±25.1	589.3±29.5	809.2±8.5	567.2±5.2	2026.1±93.2	27.8±0.9	–	–	–
HZ-9	767.6±14.4	672.5±1.8	812.7±31.4	595.8±12.8	2864.4±108.1	31.2±0.1	–	–	–
HZ-10	1029.0±10.1	734.7±7.1	754.7±3.5	585.7±20.0	3081.4±72.7	49.6±2.1	–	–	–
HZ-11	1047.8±34.2	826.7±30.1	831.6±2.8	602.3±15.4	4131.1±150.2	18.8±1.3	–	–	–
HZ-12	3069.0±26.5	1074.6±2.9	1007.5±4.3	1127.5±16.0	6118.7±37.6	20.9±1.2	–	–	–
HZ-13	820.7±11.2	540.2±6.3	887.2±18.3	587.3±22.1	2275.8±42.6	33.1±0.7	–	–	–
HZ-14	1086.3±36.3	723.6±18.8	930.3±15.2	696.7±24.5	3363.0±108.8	21.9±0.5	–	–	–
HZ-15	1037.3±36.8	827.7±14.5	671.0±20.2	547.9±23.2	5830.6±42.8	19.3±1.2	–	–	–
HZ-16	873.0±41.1	667.5±28.4	751.5±33.7	592.6±28.9	4352.4±163.6	16.6±9.0	–	–	–
YZ-1	2091.4±80.4	1506.4±21.7	1233.5±35.6	1003.2±33.1	8692.0±95.7	39.5±1.2	–	–	–
YZ-2	1482.3±15.7	885.6±18.3	798.4±15.0	575.1±20.4	4339.1±76.0	25.6±1.0	–	–	–
YZ-3	1691.4±20.6	1282.1±43.6	791.3±25.0	554.7±13.5	7594.3±344.0	24.2±1.1	–	–	–
YZ-4	714.8±20.3	476.5±5.1	686.6±14.7	449.6±7.7	2097.9±72.5	33.2±0.8	–	–	–
YZ-5	763.0±15.6	766.8±19.4	818.7±12.7	533.5±22.0	4192.3±76.9	44.1±2.0	–	–	–
YZ-6	1606.7±69.2	1142.6±26.0	773.5±24.0	577.7±20.8	6343.7±198.4	31.9±1.3	–	–	–
YZ-7	890.2±15.1	615.7±25.2	924.5±31.4	583.2±20.9	1854.5±17.3	19.5±0.8	–	–	–

Table S1 continued

ZS-1	654.5±8.2	501.6±15.9	695.8±26.5	529.4±21.5	2337.2±29.9	37.4±0.6	–	–	–
ZS-2	880.0±13.0	517.2±18.8	781.2±9.9	537.0±11.4	2027.3±52.4	32.7±1.0	–	–	–
ZS-3	464.2±8.7	285.1±6.6	783.3±18.6	608.1±23.6	909.2±28.3	49.7±1.4	–	–	–
ZS-4	710.4±5.8	509.7±14.4	745.9±14.4	557.7±15.2	2413.9±65.6	39.0±1.4	–	–	–
ZS-5	820.7±18.2	607.6±18.1	706.8±20.1	474.9±19.6	2158.7±23.2	25.0±1.3	–	–	–
ZS-6	1217.9±30.5	704.4±3.8	791.8±23.6	595.1±29.5	3027.2±66.7	25.0±0.2	–	–	–
ZS-7	1130.6±45.2	831.4±32.6	940.5±14.0	653.4±13.8	3716.5±54.5	37.8±6.0	–	–	–
GZ-1	1542.3±8.1	1049.6±24.0	788.2±13.5	562.5±8.4	5710.9±117.2	20.3±1.1	–	–	–
GZ-2	488.8±10.2	306.4±1.7	555.0±14.6	401.8±12.4	328.1±9.2	69.5±3.4	–	–	–
GZ-3	1871.2±44.5	1145.9±27.7	827.8±13.1	616.2±21.6	5891.3±154.4	13.9±0.7	–	–	–
GZ-4	310.5±6.4	112.1±1.4	816.5±7.8	589.9±30.7	–	65.1±3.3	–	–	–
GZ-5	1731.4±11.2	1042.4±21.3	838.9±20.4	669.6±20.6	4417.6±112.3	24.6±1.5	–	–	–
GZ-6	279.3±5.4	143.4±7.0	635.8±6.9	462.5±12.4	–	60.1±1.3	–	–	–
GZ-7	487.7±3.3	361.2±4.7	697.6±23.8	484.2±9.6	115.1±9.0	54.5±2.5	–	–	–
GZ-8	1548.6±29.4	1016.0±16.1	885.2±20.2	668.9±13.6	5124.4±109.1	25.4±1.6	–	–	–
BZ-1	1498.6±9.4	1009.6±14.0	720.8±27.1	656.5±17.5	5062.1±103.2	31.5±1.1	–	–	–
BZ-2	1279.0±11.3	968.2±45.4	480.2±19.9	448.7±20.5	4858.7±219.3	29.1±0.7	–	–	–
BZ-3	1307.6±44.9	1033.2±41.6	598.8±12.4	448.2±14.7	4683.0±165.8	52.7±2.4	–	–	–
BZ-4	1638.9±18.6	1226.1±33.7	617.5±11.4	465.6±14.4	6310.0±137.5	16.1±0.6	–	–	–
BZ-5	1108.9±36.8	765.0±23.8	564.6±15.9	423.8±8.8	2520.8±98.3	28.3±1.4	–	–	–
BZ-6	938.8±6.7	525.6±20.2	518.3±8.3	413.4±8.2	2478.5±66.1	56.5±2.5	–	–	–
BZ-7	1390.2±13.2	1007.3±16.9	632.1±9.9	471.9±8.9	5785.2±145.0	23.0±1.4	–	–	–

'–', Undetected

Table S2. The information of RP samples used in this study

Batch No.	Sample	Product	Diameter (mm)	Chinese herbal medicine market	Origin
1	HZ-1	Crude (phloem)	3.1±0.2	Heze (Shandong, China)	Shandong
2	HZ-2	Crude (phloem)	3.8±0.2	Heze (Shandong, China)	Shanxi
3	HZ-3	Crude (phloem)	4.1±0.2	Heze (Shandong, China)	Shanxi
4	HZ-4	Crude (phloem)	3.0±0.2	Heze (Shandong, China)	Shanxi
5	HZ-5	Crude (phloem)	3.2±0.2	Heze (Shandong, China)	Jilin
6	HZ-6	Crude (phloem)	4.5±0.2	Heze (Shandong, China)	Shanxi
7	HZ-7	Crude (phloem)	3.7±0.2	Heze (Shandong, China)	Shanxi
8	HZ-8	Crude (phloem)	2.2±0.2	Heze (Shandong, China)	Shandong
9	HZ-9	Crude (phloem)	2.9±0.2	Heze (Shandong, China)	Shanxi
10	HZ-10	Crude (phloem)	2.8±0.2	Heze (Shandong, China)	Shanxi
11	HZ-11	Crude (phloem)	3.5±0.2	Heze (Shandong, China)	Shanxi
12	HZ-12	Crude (phloem)	3.4±0.2	Heze (Shandong, China)	Shanxi
13	HZ-13	Crude (phloem)	2.8±0.2	Heze (Shandong, China)	Hebei
14	HZ-14	Crude (phloem)	3.2±0.2	Heze (Shandong, China)	Shanxi
15	HZ-15	Crude (xylem + phloem)	3.5±0.2	Heze (Shandong, China)	Gansu
16	HZ-16	Crude (xylem + phloem)	3.3±0.2	Heze (Shandong, China)	Shanxi
17	YZ-1	Crude (phloem)	2.5±0.2	Yuzhou (Henan, China)	-
18	YZ-2	Crude (phloem)	3.3±0.2	Yuzhou (Henan, China)	-
19	YZ-3	Crude (phloem)	3.4±0.2	Yuzhou (Henan, China)	-
20	YZ-4	Crude (phloem)	2.5±0.2	Yuzhou (Henan, China)	-
21	YZ-5	Crude (phloem)	4.0±0.2	Yuzhou (Henan, China)	-
22	YZ-6	Crude (phloem)	4.1±0.2	Yuzhou (Henan, China)	-

Table S2 continued

23	YZ-7	Crude (phloem)	1.8±0.2	Yuzhou (Henan, China)	-

24	ZS-1	Crude (phloem)	2.9±0.2	Zhangshu (Jiangxi, China)	-
25	ZS-2	Crude (phloem)	3.0±0.2	Zhangshu (Jiangxi, China)	-
26	ZS-3	Crude (phloem)	2.8±0.2	Zhangshu (Jiangxi, China)	-
27	ZS-4	Crude (phloem)	3.7±0.2	Zhangshu (Jiangxi, China)	-
28	ZS-5	Crude (phloem)	2.3±0.2	Zhangshu (Jiangxi, China)	Shanxi
29	ZS-6	Crude (phloem)	2.9±0.2	Zhangshu (Jiangxi, China)	Shanxi
30	ZS-7	Licorice-processed (xylem + phloem)	3.4±0.2	Zhangshu (Jiangxi, China)	Shanxi
31	GZ-1	Crude (phloem)	3.2±0.2	Guangzhou (Guangdong, China)	Shanxi
32	GZ-2	Crude (phloem)	3.2±0.2	Guangzhou (Guangdong, China)	Guizhou
33	GZ-3	Crude (phloem)	4.6±0.2	Guangzhou (Guangdong, China)	Shanxi
34	GZ-4	Crude (phloem)	3.7±0.2	Guangzhou (Guangdong, China)	Guizhou
35	GZ-5	Crude (phloem)	3.3±0.2	Guangzhou (Guangdong, China)	Shanxi
36	GZ-6	Crude (phloem)	4.0±0.2	Guangzhou (Guangdong, China)	-
37	GZ-7	Crude (phloem)	2.2±0.2	Guangzhou (Guangdong, China)	-
38	GZ-8	Crude (phloem)	3.9±0.2	Guangzhou (Guangdong, China)	Shanxi
39	BZ-1	Licorice-processed (xylem + phloem)	4.3±0.2	Bozhou (Anhui, China)	Guizhou
40	BZ-2	Crude (xylem + phloem)	4.1±0.2	Bozhou (Anhui, China)	Guizhou
41	BZ-3	Licorice-processed (xylem + phloem)	3.3±0.2	Bozhou (Anhui, China)	Shanxi
42	BZ-4	Crude (xylem + phloem)	4.0±0.2	Bozhou (Anhui, China)	Shanxi
43	BZ-5	Crude (xylem + phloem)	2.9±0.2	Bozhou (Anhui, China)	Hubei
44	BZ-6	Licorice-processed (xylem + phloem)	3.2±0.2	Bozhou (Anhui, China)	Shanxi
45	BZ-7	Crude (xylem + phloem)	3.5±0.2	Bozhou (Anhui, China)	Shanxi

'–', Unknown
